# *Borrelia burgdorferi* Promotes the Establishment of *Babesia microti* in the Northeastern United States

**DOI:** 10.1371/journal.pone.0115494

**Published:** 2014-12-29

**Authors:** Jessica M. Dunn, Peter J. Krause, Stephen Davis, Edouard G. Vannier, Meagan C. Fitzpatrick, Lindsay Rollend, Alexia A. Belperron, Sarah L. States, Andrew Stacey, Linda K. Bockenstedt, Durland Fish, Maria A. Diuk-Wasser

**Affiliations:** 1 School of Mathematical and Geospatial Sciences, RMIT University, Melbourne, Australia; 2 Department of Epidemiology of Microbial Diseases, Yale School of Public Health, New Haven, Connecticut, United States of America; 3 Department of Internal Medicine, Yale School of Medicine, New Haven, Connecticut, United States of America; 4 Division of Geographic Medicine and Infectious Diseases, Tufts Medical Center, Boston, Massachusetts, United States of America; 5 Department of Ecology, Evolution and Environmental Biology, Columbia University, New York, New York, United States of America; The University of Texas at San Antonio, United States of America

## Abstract

*Babesia microti* and *Borrelia burgdorferi*, the respective causative agents of human babesiosis and Lyme disease, are maintained in their enzootic cycles by the blacklegged tick (*Ixodes scapularis*) and use the white-footed mouse (*Peromyscus leucopus*) as primary reservoir host. The geographic range of both pathogens has expanded in the United States, but the spread of babesiosis has lagged behind that of Lyme disease. Several studies have estimated the basic reproduction number (*R*_0_) for *B. microti* to be below the threshold for persistence (<1), a finding that is inconsistent with the persistence and geographic expansion of this pathogen. We tested the hypothesis that host coinfection with *B. burgdorferi* increases the likelihood of *B. microti* transmission and establishment in new areas. We fed *I. scapularis* larva on *P. leucopus* mice that had been infected in the laboratory with *B. microti* and/or *B. burgdorferi*. We observed that coinfection in mice increases the frequency of *B. microti* infected ticks. To identify the ecological variables that would increase the probability of *B. microti* establishment in the field, we integrated our laboratory data with field data on tick burden and feeding activity in an *R*_0_ model. Our model predicts that high prevalence of *B. burgdorferi* infected mice lowers the ecological threshold for *B. microti* establishment, especially at sites where larval burden on *P. leucopus* is lower and where larvae feed simultaneously or soon after nymphs infect mice, when most of the transmission enhancement due to coinfection occurs. Our studies suggest that *B. burgdorferi* contributes to the emergence and expansion of *B. microti* and provides a model to predict the ecological factors that are sufficient for emergence of *B. microti* in the wild.

## Introduction

Interactions between pathogens in multiply infected hosts strongly influence pathogen virulence, transmission and persistence [1]–[11]. Tick-borne infections offer an attractive model system to study pathogen interactions because multiple pathogens are known to co-exist in ticks and vertebrate reservoir hosts [12]–[24]. At least six emerging pathogens are transmitted from *Ixodes scapularis* ticks to their natural reservoir hosts and to humans, including *Borrelia burgdorferi* sensu stricto (Lyme disease), *Babesia microti* (babesiosis), *Anaplasma phagocytophilum* (anaplasmosis), *Borrelia miyamotoi* (“hard tick relapsing fever”), Powassan virus (Powassan virus disease) and *Ehrlichia muris*-like pathogen [7], [21], [25]–[27]. *B. burgdorferi* is transmitted more efficiently than the other five pathogens, has followed *I. scapularis* geographic expansion during the past three decades, and is highly prevalent in most *I. scapularis* populations in the northern United States [28]–[30]. As other *I. scapularis*-borne pathogens are introduced into areas enzootic for *B. burgdorferi*, co-infections in hosts and ticks may modify the dynamics of transmission and propagation of these pathogens.

Although *B. burgdorferi* and *B. microti* are transmitted by the same vector in the northeastern and upper midwestern regions of the United States, the geographic spread of babesiosis has lagged behind that of Lyme disease [25], [31]–[35]. The delayed expansion of *B. microti* has been attributed to a lower efficiency of transmission between *Peromyscus leucopus* (white-footed mouse) and ticks [30] and to a narrower range of vertebrate reservoir hosts when compared with *B. burgdorferi* [36]. These observations are consistent with the lower basic reproduction number (*R*_0_) reported for *B. microti* compared to that of *B. burgdorferi.* In fact, the *B. microti R*_0_ has been estimated to be lower than the threshold for pathogen persistence (<1), raising the question of how it persists and expands in the northeastern United States [37], [38].

Given that the establishment of *B. burgdorferi* typically precedes that of *B. microti*, we tested the hypothesis that coinfection of hosts with *B. burgdorferi* enhances the likelihood of *B. microti* establishment. To do so, we assessed the effect of coinfection at the individual host level in a laboratory setting that replicates pathogen-tick-host interactions that exist in the field. We then extended this observation to the population level by use of a mathematical model and identified ecological thresholds for *B. microti* establishment (*R*_0_>1). This model was made ecologically realistic by using data on ecological parameters obtained from two field sites in southern New England that are epidemiologically and ecologically distinct.

## Materials and Methods

### Laboratory infection experiments

#### Sources of mice and ticks

*P. leucopus* mice (LL stock) were obtained from the University of South Carolina *Peromyscus* Genetic Stock Center and housed in a Yale Animal Resource Center facility. All procedures were approved by the Yale Institutional Animal Care and Use Committee (Protocol #07689). Mice were exposed to a diurnal light-dark cycle (16L:8D) and singly housed on wire cage bottoms over water to allow for collection of replete ticks. Mice were anesthetized prior to each infestation with nymphal or larval ticks. Infected *I. scapularis* nymphs were produced by allowing uninfected *I. scapularis* larvae to feed to repletion on infected mice. Fed larvae were collected and maintained in environmental chambers set at 21°C and >90% relative humidity. After molting, nymphs were stored at 8°C and in >90% relative humidity until experimental infestations. Uninfected *I. scapularis* larvae used for xenodiagnoses were produced by feeding wild-collected adult female *I. scapularis* on New Zealand White rabbits (Charles River Laboratories, Inc.); replete females were stored at 8°C and in >90% relative humidity until they laid eggs, and moved to 21°C and in >90% relative humidity until the hatching of eggs.

#### Infection of *P. leucopus* mice with *B. microti* and *B. burgdorferi*

**Figure 1:**
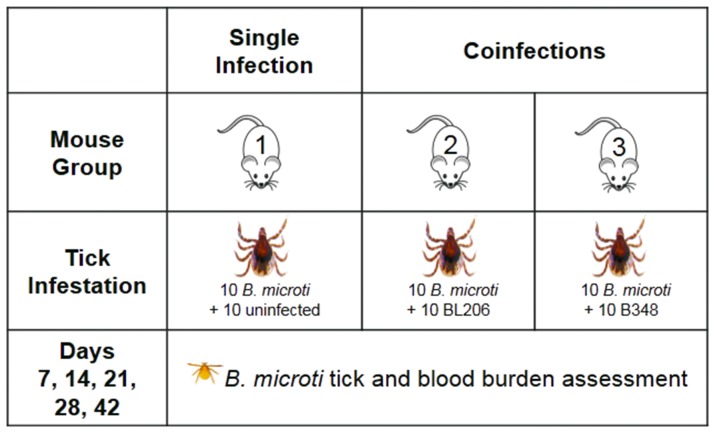
Laboratory study design. *Peromyscus leucopus* mice were infected with *Babesia microti* alone (Group 1 [8 mice]) or coinfected with *B. microti* and one of two strains of *Borrelia burgdorferi:* BL206 (Group 2 [3 mice]) or B348 (Group 3 [8 mice]). Xenodiagnosis was performed at 7, 14, 21, 28, 42 days. *B. microti* infection was determined in ticks at 7, 14, 21, 28, 42 days by qPCR. *B. microti* infection was determined in mouse blood at weeks 7, 14, 28, 42 days by flow cytometry.

The experiment was carried out in two sets. In the first set (Fig. 1), *P. leucopus* mice were infested with nymphal ticks infected with *B. microti*, or a combination of nymphal ticks infected with *B. microti* and either the *B. burgdorferi* strain BL206 or the *B. burgdorferi* strain B348. The *B. microti* strain was previously isolated from a *Peromyscus leucopus* mouse trapped in Greenwich, CT and maintained by alternate passaging between C3H/HeJ *Prkdc^scid^* mice and *I. scapularis* ticks [31]. The two *B. burgdorferi* strains have polarized infectious phenotypes. BL206 is characterized by an *Osp* C genotype A and is highly invasive; it migrates from the skin into the bloodstream and reaches secondary sites. In contrast, B348 is characterized by an *Osp* C genotype E and is non-invasive as it remains at the tick bite site [39]–[46]. In the second set of experiments, mice were infected with *B. microti* alone or together with *B. microti* and B348. Data from these two sets were combined for statistical analyses.

#### Assessment of *B. microti* transmission from *P. leucopus* mice to ticks

We used xenodiagnoses to assess pathogen transmission from mice to ticks, as previously described [47], [48]. One hundred uninfected larval ticks were placed on each mouse on days 7, 14, 21, 28 and 42 post nymphal infestation. Fed larvae were collected from water trays placed beneath the mouse cages and maintained in environmental chambers until they molted into nymphs. The *B. microti* burden was assessed in 20 nymphs that were randomly selected among those obtained from each mouse.

#### Detection of *B. microti* DNA in ticks

Individual nymphal ticks that had been stored frozen in liquid nitrogen were homogenized using sterile pestles. DNA was extracted using the DNeasy Blood and Tissue Kit (QIAGEN, Valencia CA), and eluted in 120 µL of 10 mM Tris·HCl at pH 8.5. The *B. microti* 18S rRNA gene (GenBank accession number AY144696.1) was amplified by quantitative PCR [49] using these forward and reverse primers and probe (from 5′ to 3′): AACAGGCATTCGCCTTGAAT, CCAACTGCTCCTATTAACCATTACTCT, and 6FAM-CTACAGCATGGAATAATGA-MGBNFQ, respectively. The PCR reaction consisted of 2X Taqman Universal PCR Master Mix (with AmpErase, Applied Biosystems, Foster City CA), 0.9 µM forward and reverse primers, 0.2 µM probe, and 5 µL DNA template in a total reaction volume of 25 µL. DNA was amplified in an Applied Biosystems 7500. Ticks were considered positive for *B. microti* DNA if amplicons were detected at or below a cycle threshold (C_T_) value of 35 [49].

#### Assessment of *B. microti* parasitemia in mice

On days 7, 14, 28, and 42 after nymphal infestation of *P. leucopus* mice, 50 µL of peripheral blood were obtained via submandibular venipuncture. Blood cells were fixed in glutaraldehyde, permeabilized in Triton X-100, and treated with DNase-free RNase, as previously described [50]. Parasites were detected using the nucleic acid dye YOYO-1 iodide. For each mouse blood sample, 10,000 cells were acquired using the FACS Calibur (Becton Dickinson, San Jose, CA). Upon excitation by the argon-ion laser (488 nm), fluorescence emission (509 nm) was recorded using CellQuest (Scripps Research Institute, La Jolla, CA) and analyzed using FlowJo (TreeStar, Ashland, OR).

#### Statistical analyses

We examined whether coinfection of mice with *B. microti* and *B. burgdorferi* increases the probability of nymphal infection with *B. microti*, when compared with mice infected with *B. microti* alone. Statistical analyses were performed using generalized estimating equation population-averaged logit models in STATA/SE, version 12.0 (STATA Corporation, College Station, TX). These generalized linear models allow specification of the within-group (panel) correlation structure. Because multiple ticks were allowed to feed on an individual mouse, a within mouse correlation structure was specified in the model to account for the autocorrelation in the infection status among the ticks that fed on the same mouse. We treated time as a continuous variable (days since infection with *B. microti* and/or *B. burgdorferi*) and assessed interactions between mouse group and time. Differences in parasitemia among groups were assessed using negative binomial regression, including a term to account for days since infection with *B. microti*.

### Field sampling

To obtain realistic estimates of ecological parameters as inputs for modeling of *B. microti* emergence, we determined the tick burdens and the seasonal pattern of tick feeding (phenology) on *P. leucopus* mice trapped at two ecologically distinct sites: eastern Connecticut (Nehantic and Pachaug State Forests) and Block Island, Rhode Island (Old Mill, Rodman's Hollow, and West Beach). The host community on Block Island is dominated by *P. leucopus* and is less diverse than that at the Connecticut sites. Furthermore, the tick burdens are higher at the Block Island sites, when compared with the mainland sites [51]. Trapping was conducted in

Nehantic State Forest in Lyme, CT (41.391531, −72.301304) (22 May to 15 August 2013), and in Pachaug State Forest in North Stonington, CT (41.493028, −71.853611) (29 May to 22 August 2013). At both sites, 144 traps were set 10 m apart in a 12×12 grid formation (total area of the grid: 14,400 m^2^). Trapping on Block Island was conducted at three forested sites: Old Mill (41.163213, −71.589958) (29 May to 8 August 2013), Rodman's Hollow (41.151258, −71.588489) (21 May to 2 August 2013), and West Beach (41.210015, −71.572009) (29 May to 8 August 2013). The Old Mill and West Beach trapping grids consisted of 60 traps (6×10 grid) and 58 traps (irregular grid), respectively, whereas the Rodman's Hollow site had 120 traps (10×12 grid). At every site, trapping occurred bimonthly for three consecutive nights per session for a total of seven sessions at the Connecticut sites and six sessions at the Block Island sites. *P. leucopus* mice were live-trapped in Sherman box traps (9″×3″×3.5″) which were baited with rolled oats, sunflower seeds and cotton balls, set at dusk, and checked shortly after dawn the next day. Captured individuals were ear-tagged, aged, sexed, weighed, searched for ticks, bled by submandibular venipuncture, and subjected to ear punch biopsy before release at the point of capture. Ticks were removed with forceps and preserved in 70% ethanol. All field study procedures were approved by the Yale Institutional Animal Care and Use Committee (Protocol #07596). Inhalation of isoflurane was the approved method of euthanasia for situations when warranted, however, no animals had to be euthanized during this study. Field studies did not involve endangered or protected species. Property access permissions and scientific collector's permits were obtained from the Connecticut Department of Energy and Environmental Protection, the Rhode Island Department of Environmental Management, The Nature Conservancy, and the US Fish & Wildlife Service (Charlestown, RI).

### Mathematical *(R*_0_ *)* modeling

**Figure 2:**
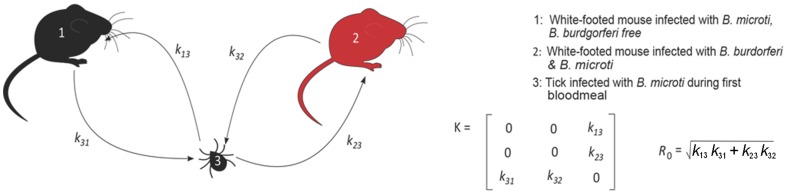
The transmission graph for *Babesia microti* for singly infected and coinfected mice. Three ‘host types’ are defined: (1) *Peromyscus leucopus* mouse, *Borrelia burgdorferi* free, infected with *B. microti*, (2) *P. leucopus* mouse infected with *B. burgdorferi* and *B. microti* and (3) tick infected with *B. microti* during first blood meal. The transmission graph is used in the construction of the next-generation matrix (*K*) for *R*_0_ where *k_ij_* indicates the expected number of host type *i* infected by host type *j*.

To determine whether host coinfection significantly increases the likelihood of *B. microti* establishment in the wild, we integrated laboratory and field data into a mathematical model that estimates the basic reproduction number *R*_0_. The basic reproduction number describes the *expected* initial spread of a pathogen that arrives in a naïve host population and is used to predict the ecological conditions that allow pathogen establishment (parameter ranges for which *R*_0_>1) or result in pathogen fade-out (parameter ranges for which *R*_0_<1) [52]. Larger *R*_0_ values also imply an increased likelihood of pathogen establishment and therefore a shorter time to establishment. Our model is derived from the model constructed by Dunn et al. (2013) [38], which was based on the original model by Hartemink et al. (2008) [53] and modified by Davis and Bent (2011) [37]. These models assume that *R*_0_ is a function of one *B. microti* infected nymph or mouse introduced into an already established *B. burgdorferi* infected population. Parameters used in the *R*_0_ calculation were estimated from our laboratory and field experiments (see above) and from the literature. Parameter definitions and parameter point values for the Connecticut and Block Island sites are shown in Table 1 [37], [38], [54]–[61]. Based on serological studies of mammals trapped in the wild, we considered two prevalence rates for *B. burgdorferi* infection in *P. leucopus* mice: a high rate of 0.80 and a low rate of 0.30 [12], [62], [63]. Additional information, including full parameter ranges and the results of a global sensitivity analysis of *R*_0_, is published elsewhere [38]. Based on evidence of sequential geographic expansion of *B. burgdorferi* and *B. microti* [25], [34], our model assumes that *B. microti* is spreading into new sites where *B. burgdorferi* is already enzootic. The formulation assumes that a constant fraction (γ) of *P. leucopus* mice is infected with *B. burgdorferi* (Fig. 2), thereby defining three types of hosts/vectors: (1) a *P. leucopus* infected with *B. microti*, (2) a *P. leucopus* coinfected with *B. burgdorferi* and *B. microti* and, (3) a tick infected during its first blood meal. The model was restricted to *P. leucopus* mice because they are the main vertebrate hosts for *B. microti* [36] and because laboratory transmission efficiency data are available for this species; however, our modeling does take into account that a proportion of the tick population, 1-*c*, feeds on other hosts (Table 1).

**Table 1:** Definitions of parameters appearing in the *R*_0_ model and point parameter values for the Connecticut and Block Island populations.

Function	Parameter	Parameter Description	Connecticut Population Point Value	Block Island Population Point Value	Data source
	*d_L_*	Days of attachment for larvae [days]	4	4	37, 54, 55
	*s_N_*	Survival from infected larva to infected nymph [days]	0.40	0.40	37, 56
	*q_N_*	Probability of nymph to mouse transmission	0.83	0.83	37, 57
	*c*	Proportion of ticks feeding on *P. leucopus*	0.50	0.50	
*θ*	*ρ*	Mean survival of mice	133	133	58, 59
	μ*_N_*	Time between the beginning of nymphal activity and peak nymphal activity [days]	35.07	25.49	Field data, 60
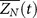	τ*_N_*	Timing of beginning of nymphal activity [days]	124.73	134.70	Field data, 60
	σ*_N_*	Shape parameter nymphal activity	0.62	0.52	Field data, 60
	*H_N_*	Height nymphal activity	0.60	5.96	Field data, 60
	μ*_E_*	Time between the beginning of larval activity and first larval peak [days]	24.41	39.01	Field data, 60
	μ*_L_*	Time between the beginning of second period of larval activity and second larval peak [days]	59.67	39.01	Field data, 60
	τ*_E_*	Timing of beginning of first larval activity [days]	160.06	155.31	Field data, 60
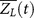	τ*_L_*	Timing of beginning of second larval activity [days]	171.40	185.44	Field data, 60
	σ*_L_*	Shape parameter second larval peak	0.26	0.59	Field data, 60
	*H_E_*	Height first larval peak [days]	4.63	0.36	Field data, 60
	*H_L_*	Height second larval peak [days]	10.48	33.38	Field data, 60

A full description of parameters and corresponding references and field data can be found in [38]. Burden parameters assume January 1^st^ as day 0.

The next-generation matrix is constructed according to the transmission probabilities in Fig. 2 with the *k_ij_* defined as the expected number of host type *i* infected by a single individual of host type *j* over its entire infectious period (a zero entry indicates transmission from host type *j* to host type *i* is negligible). *R*_0_ is then the dominant eigenvalue of the next-generation matrix (Fig. 2). The functional forms of *k_23_*, *k_32_*, *k_13_* and *k_31_* are adapted from [53] where *k_23_* and *k_13_* are, respectively, the expected number of uninfected *P. leucopus* infected with *B. microti* from a nymphal tick infected with *B. microti*, and the expected number of *B. burgdorferi*-infected *P. leucopus* infected with *B. microti* from a nymphal tick infected with *B. microti*. *R*_0_ takes the form
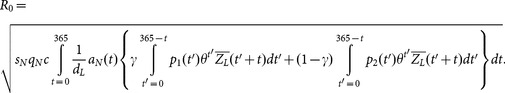
(1)


Here, *s_N_, q_N_*, *c* and *d_L_*, respectively, represent the probability of survival from infected fed larva to infectious feeding nymph, the probability of transmission from nymph to mouse, the proportion of ticks feeding on *P. leucopus*, and the duration of tick attachment for larvae taking a blood meal. The parameter *θ* takes into account mouse survivorship with lifespan *ρ* and is given by,
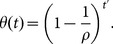
(2)


The functions 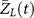 and *a_N_*(*t*) are, respectively, the mean larvae burden and the scaled mean nymphal burden on *P. leucopus* and are given by,
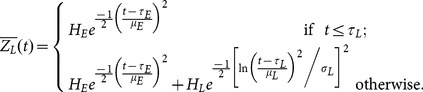
(3)and,
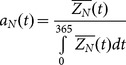
(4)with 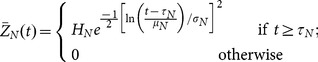
(5)


The phenology curves, 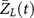 and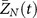, have well defined parameters for the heights of each peak, timing of beginning of activity and time to peak activity (S1 Fig.). There is an additional shape parameter which controls the tail of the distributions. These curves were fitted to our field data consisting of larval and nymph counts from trapped mice. The data were pooled for the two sites in Connecticut and the three sites on Block Island, Rhode Island using the same combinations of normal and right-shifted log-normal functional forms as previously published [60]. These curves represent the phenology of the immature life stages of *I. scapularis* as observed in areas of the northeastern and upper midwestern United States where both *B. burgdorferi* and *B. microti* are endemic. The functional forms used are informed by a large number of field studies that show an initial normal peak in questing larvae in late spring followed by a larger second log-normal peak. Nymphal activity is well represented by a single, log-normal curve [60]. Finally, the efficiency of transmission of *B. microti* from mice singly infected with *B. microti* and from mice coinfected with *B. microti* and *B. burgdorferi*, as measured from the laboratory experiments (see Results), are included by constructing functions, *p*_1_(*t*) and *p*_2_(*t*). These are piecewise linear functions fitted to the transmission efficiencies observed at 7, 14, 21, 28 and 42 days after infection. Transmission efficiency is assumed to simply change linearly between these observed values. The 95% confidence intervals were constructed by using bootstrap methods [64].

## Results

### Pathogen interactions in the laboratory setting: the frequency of *B. microti* infected nymphs increases when they feed as larvae on *P. leucopus* mice coinfected with *B. burgdorferi*

**Figure 3:**
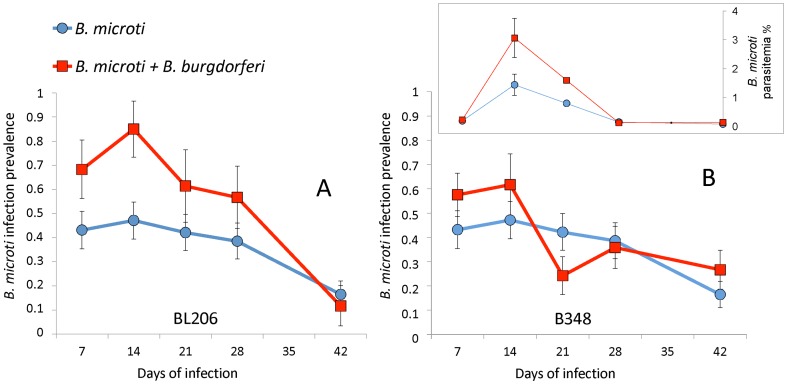
Effect of coinfection on larval acquisition of *Babesia microti*. The results show the prevalence of *B. microti* in xenodiagnostic ticks that fed on mice infected with *B. microti* alone compared to mice coinfected with *B. microti* and *Borrelia burgdorferi* strain BL206 (A) or *B. burgdorferi* strain B348 (B). *B. microti* parasitemia in mice infected with *B. microti* alone or *B. microti* and *B. burgdorferi* strain B348 are shown in inset above B. The error bars indicate 95% confidence intervals.

We determined the effect of *B. burgdorferi* host coinfection on the acquisition of *B. microti* by ticks. The frequency of *B. microti* infected nymphs was higher when larvae fed on mice coinfected with *B. microti* and the highly invasive *B. burgdorferi* strain BL206 (Group 2, Fig. 1) than on mice infected with *B. microti* alone (Group 1, Fig. 1) (odds ratio  = 3.73, p<0.05) (Fig. 3A, S1 Table). The frequency of *B. microti* infected nymphs declined over time following infection of the host, as indicated by a significant effect of days post-infestation on the percentage of infected ticks (odds ratio  = 0.95, p<0.001) (Fig. 3A, S1 Table).

Nymphs derived from larvae that had fed on mice coinfected with *B. microti* and the non-invasive *B. burgdorferi* strain B348 (Group 3, Fig. 1) were as frequently infected with *B. microti* as those derived from larvae that had fed on mice infected with *B. microti* alone (Group 1, Fig. 1 and Fig. 3B). Given that the frequency of *B. microti* infected ticks appeared to increase during the first two weeks after infection, we next compared the frequency of infection in ticks that had fed on coinfected and non-coinfected mice during this short time period. During the first 14 days post-infection, coinfection significantly increased the frequency of *B. microti* infected nymphs (odds ratio  = 4.6, p = 0.045).

We determined whether the higher frequency of *B. microti*–infected nymphs was associated with a higher *B. microti* parasitemia in mice. Parasitemia peaked on day 14 whether mice were coinfected with B348 and *B. microti* or with *B. microti* alone (Fig. 3B inset). Parasitemia was higher in the coinfected group than in the *B. microti* only group (negative binomial regression, LR Chi2  = 23.4, p<0.001), consistent with the higher frequency of *B. microti*–infected nymphs in the coinfected group. *B. microti* parasitemia was not monitored in mice coinfected with BL206.

### Ecological parameters influencing pathogen transmission in the field: higher tick burdens on Block Island but greater synchrony of larval and nymphal feeding in Connecticut

**Figure 4:**
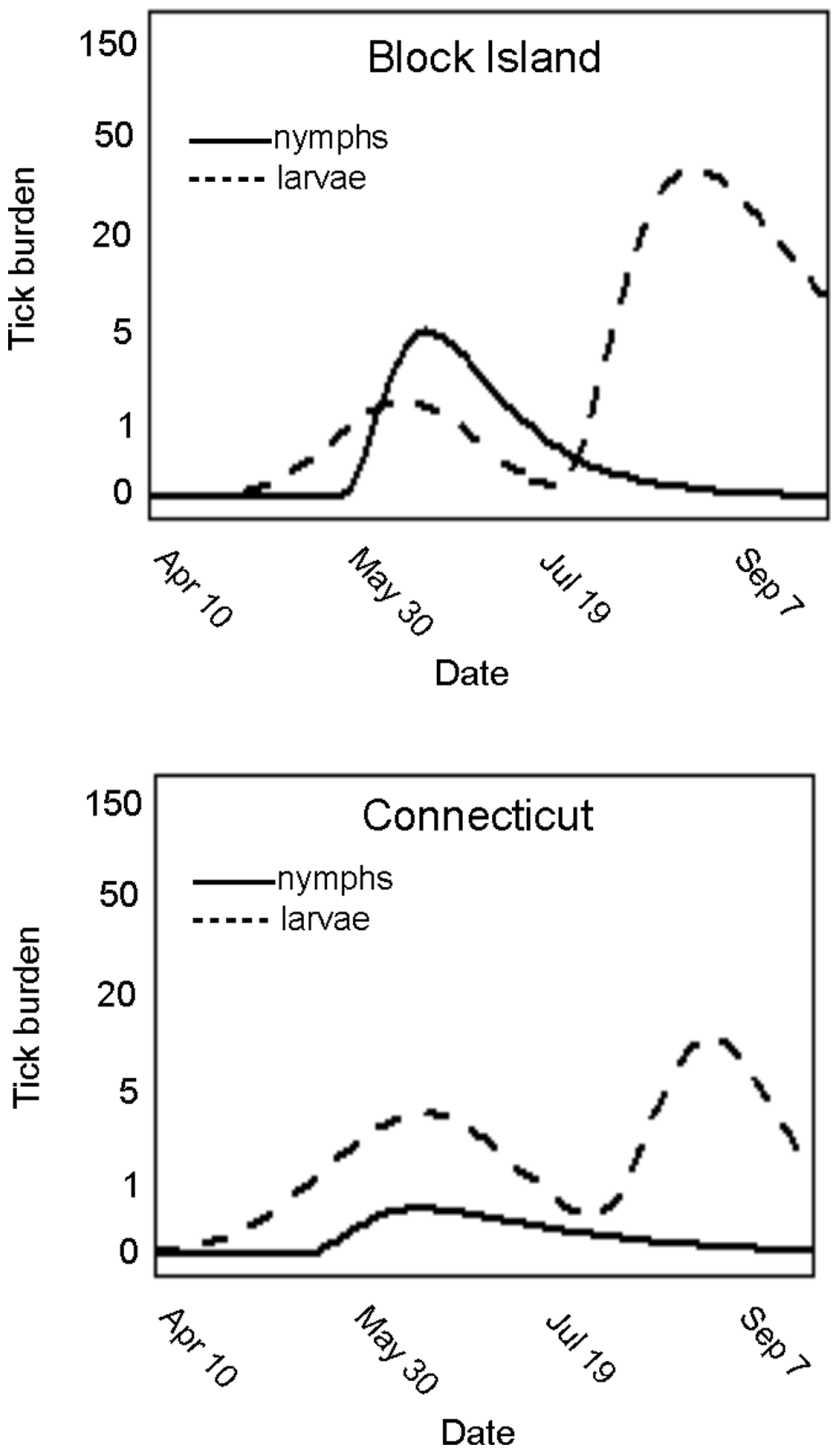
Larval and nymphal tick burdens on *Peromyscus leucopus* expressed as mean tick count per mouse. Tick burdens are presented as best fit curves to field derived data from Block Island, RI (top) and Connecticut (bottom) sampled populations (S2 Fig.).

We examined factors that would enhance pathogen transmission at our study sites. We quantified the tick burdens on mice trapped at sites in eastern Connecticut and on Block Island, Rhode Island. Larval and nymphal tick burdens were higher on mice at the Block Island sites than on those trapped in Connecticut (Fig. 4, S2 Fig.). At both sites, nymphal activity peaked in late spring (June 9 in both Connecticut and Block Island). The first peak of larval activity also occurred in late spring (June 4 in Connecticut; June 13 on Block Island) and overlapped with nymphal activity whereas the second peak was reached in mid to late summer (August 19 in Connecticut; August 12 on Block Island) (Fig. 4, S2 Fig.). The more intense spring larval peak in Connecticut significantly overlapped with the nymphal activity peak and thus provided *B. microti* transmission opportunities during the early stages of infection when there was a stronger effect of coinfection on transmission efficiency (Fig. 3 and Fig. 4). In contrast, most larval activity occurred in late summer on Block Island, resulting in a small overlap with the tail end of the spring nymphal activity, i.e., when transmission efficiency is no longer enhanced by coinfection (Fig. 4).

### Integration of laboratory-derived infection parameters with field-derived tick burdens: co-infection of reservoir hosts with *B. burgdorferi* lowers the ecological requirements for *B. microti* establishment

**Figure 5:**
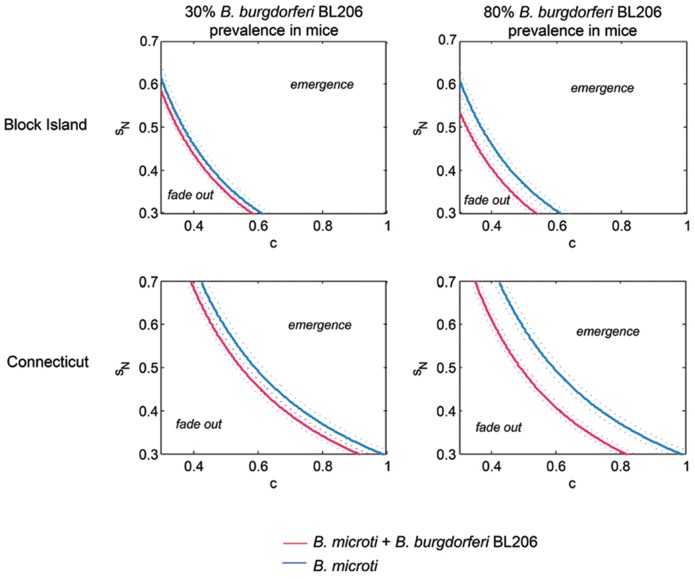
Threshold curves for *Babesia microti* survival at different locations and mouse infection prevalences with *Borrelia burgdorferi* strain BL206. This figure shows differences in threshold curves, representing where *R*_0_ = 1, and associated 95% confidence intervals (dotted curves), that separate regions of *s_N_* and *c* where *B. microti* is expected to emerge and regions where it is expected to fade out. Threshold curves are contour curves where *R*_0_ is plotted as a function of two variables: the proportion of fed infected larvae that survive to become infectious feeding nymphs, *s_N_*, and the proportion of ticks feeding on *Peromyscus leucopus*, *c.* Plots indicate effects of location specific (Block Island and Connecticut) timing of tick activity as well as *B. burgdorferi* strain BL206 strain prevalence in mice (low  = 0.3 and high  = 0.8) on *R*_0_. Coinfection significantly enhances the likelihood of *B. microti* establishment in Connecticut and on Block Island when *B. burgdorferi* prevalence among *P. leucopus* is high (0.80) (confidence bounds do not overlap). Differences in threshold curves for *B. burgdorferi* strain B348 are shown in S3 Fig.

We integrated laboratory and field data into a mathematical model that estimates the basic reproduction number *R*_0_. Based on serological studies of mammals trapped in the wild, we considered two point prevalences for *B. burgdorferi* infection in *P. leucopus* mice: a high prevalence of 0.80 and a low prevalence of 0.30 [12], [62], [63]. *R*_0_ values are presented as a function of the proportion of infected larvae that survive and molt to become feeding infectious nymphs (*S_N_*) and of the proportion of larval ticks feeding on *P. leucopus* (*c*) (Fig. 5, S3 Fig.). These two parameters were chosen on the basis of previous work [38] that ranked them as two of the most important determinants of *R*_0_, although both were ranked *below* the parameters governing pathogen transmission efficiency that were measured directly in the present study [38]. The effect of co-infection with a disseminating strain of *B. burgdorferi* (BL206) is presented in Fig. 5 whereas the effect of co-infection with a non-disseminating strain (B348) is presented in S3 Fig. Curves were fitted to the experimental infection data (see Methods) by considering nymphs fed as larvae on co-infected mice (red curves) and those fed as larvae on singly infected mice (blue curves). The larger the area above the curve (‘emergence’), the broader the conditions that allow for the establishment of *B. microti*. Taking into account 95% confidence intervals (calculated from repeated resampling of the data presented above and represented by dotted curves in Fig. 5), we concluded that coinfection significantly enhances the likelihood of *B. microti* establishment in Connecticut (increase in *R*_0_ of 15%) and on Block Island (increase in *R*_0_ of 11%) when *B. burgdorferi* prevalence among *P. leucopus* is high (0.80) (confidence bounds in Fig. 5 right panels do not overlap).

Lastly, we estimated the threshold of *B. burgdorferi* prevalence in *P. leucopus* above which *R*_0_ values are significantly increased by coinfection as determined by no overlap between the 95% confidence intervals for all values of *S_N_* and *c*. This threshold was 0.42 for the Block Island sites and 0.25 for the Connecticut sites.

## Discussion

We have demonstrated in the laboratory that the frequency of *B. microti*-infected ticks is higher when fed on *P. leucopus* that are coinfected with *B. burgdorferi* and *B. microti* than on mice infected with *B. microti* alone. In field studies, we quantified two important ecological parameters that affect pathogen transmission, tick burdens on *P. leucopus* and timing of tick feeding. By use of a mathematical model that integrates laboratory and field derived data, we identified ecological conditions that synergize with co-infection to enhance *B. microti* establishment (*R*_0_>1). Our modeling results indicate that high prevalence of *B. burgdorferi* in *P. leucopus* significantly lowers the ecological thresholds for enzootic establishment of *B. microti* at mainland and island sites, with a stronger effect at mainland sites. These results suggest that the geographic spread of *B. microti* is favored by prior enzootic establishment of *B. burgdorferi*.

The effects of coinfection on the *I. scapularis*-borne pathogen infection cycle vary according to pathogen species and genotype, as observed in previous studies of *B. microti* and of the interactions between *B. burgdorferi* and *A. phagocytophilum* [30], [47], [48]. The two *B. burgdorferi* strains used in this study had markedly different phenotypes in regard to host invasiveness and duration of infection. The proportion of *B. microti*-infected nymphs was significantly increased when mice were coinfected with the highly invasive *B. burgdorferi* strain BL206. This enhancement lasted over the initial four week study period. We also observed increased nymphal infection when larvae had fed on mice infected with both *B. microti* and the non-invasive *B. burgdorferi* strain B348, but this effect was restricted to the first two weeks of infection. Consistent with this observation, *B. microti* parasitemia was transiently higher in mice coinfected with the B348 strain than with *B. microti* alone. The highly invasive *B. burgdorferi* strain BL206 can be isolated from human blood and can be transmitted from mice to ticks for about 80 days post-infection [41], [44]–[46], [65]–[67]. In contrast, the non-invasive *B. burgdorferi* B348 strain often is found in human skin without dissemination and is efficiently transmitted from mice to ticks for only approximately 40 days [41], [44]–[46]. The difference in dissemination between *B. burgdorferi* strains may contribute to the difference in their ability to promote *B. microti* transmission, as the immune response elicited by these strains likely differ in nature and possibly duration. By remaining in the dermis at the site of a tick bite, non-invasive *B. burgdorferi* strains may be poorly able to modulate the *B. microti* specific immune response that develops in the spleen [15], [68]. In contrast, dissemination of a *B. burgdorferi* strain through the bloodstream may significantly impair the immune response required to control and eradicate *B. microti* in the host [15], [69]–[71]. Although our experiments were restricted to two representative strains, the *B. burgdorferi* strain phenotypes are likely to be relevant to *P. leucopus* infections in the field. Indeed, the monophyletic clade to which *B. burgdorferi* strain BL206 belongs [39] is highly prevalent throughout the northeastern United States [72] and strains that clustered with the BL206 strain are likely to share the invasive phenotype.

The increased proportion of *B. microti*-infected nymphs derived from larval feeding on *B. burgdorferi* coinfected mice may help explain geographic differences in endemicity and the pattern of emergence of human babesiosis. *B. burgdorferi* has expanded more rapidly than *B. microti*, presumably because it is transmitted more efficiently between ticks and white-footed mice [30]. Our observation that host coinfection increases the proportion of *B. microti* infected nymphs may help explain the high *B. microti* prevalence rates in some areas that have long been endemic for *B. burgdorferi* [25], [33]. Our modeling results show that the larger tick burdens observed on *P. leucopus* on Block Island lower the predicted ecological thresholds for *B. microti* establishment. This is consistent with the high endemicity of *B. microti* on New England coastal islands. In contrast, lower tick burdens result in higher ecological thresholds for *B. microti* establishment at the Connecticut sites. These thresholds may be reduced by coinfection when a large proportion of white-footed mice are infected with *B. burgdorferi*. Thus, the delay in *B. microti* establishment in Connecticut and other mainland sites may be attributed to the time required for establishment of *B. burgdorferi* [73], [74].

The effect of coinfection on *B. microti* establishment also depends on the timing of feeding by nymphs and larvae. *I. scapularis*-borne pathogens typically are maintained in their enzootic cycles by sequential transmission from infected nymphs to white-footed mice to uninfected larvae. A short time gap between the feeding by nymphs and the feeding by larvae increases the chance for completion of the enzootic cycle because the reservoir host is less likely to die or to mount an immune response that could eliminate the pathogen. In the northeastern United States, nymphs typically feed from late spring to early summer whereas larvae feed in two periods, one in late spring and the second one in late summer [75]. The magnitude and duration of the two larval feeding periods vary between and within endemic areas [72], [76]. White-footed mice are more likely to become infected with *B. burgdorferi* and *B. microti* from late spring to early summer, i.e., when infected nymphs transmit both pathogens during their blood meal. Given that the *B. microti* parasitemia in white-footed mice is high and the transmission of *B. microti* to feeding larvae more likely during the first two weeks after infestation in experimental mice, we expect the transmission efficiency to feeding larvae in the wild to be highest during or soon after the first larval feeding period. In this context, the intense and prolonged larval questing activity seen in late spring in Connecticut may synergize the effect of coinfection on *B. microti* establishment as revealed by an increased *R*_0_. In contrast, the more limited impact of coinfection on *B. microti* establishment on Block Island is consistent with an intense larval feeding activity in late summer. On Block Island the effect of coinfection on *B. microti* transmission fades as the summer progresses but *B. microti* transmission most likely remains high because of the large tick burden of *P. leucopus* mice. Synchronous feeding previously has been shown to play a key role in the maintenance of flaviviruses causing tick-borne encephalitis in Europe [77] and in the United States [78]. We demonstrate that it also plays a key role in the maintenance and potential for interaction of pathogens with short infectious periods. Similar to our Connecticut study sites, spring larval activity is intense in the upper Midwest [72], thereby raising the likelihood of increased *B. microti* transmission by concurrent *B. burgdorferi* infection in vertebrate hosts.

In summary, we have observed increased *B. microti* transmission from *B. burgdorferi* coinfected *P. leucopus* mice to *I. scapularis* ticks in the laboratory. We found that the strength of the coinfection effect depends on the *B. burgdorferi* strain, the tick burden on the primary vertebrate host (*P. leucopus*), and the overlap between nymphal and larval feeding periods. We incorporated these factors in a mathematical model to predict establishment of *B. microti* in a region. The model predicted that coinfection enhancement is stronger in Connecticut than on Block Island, which may partly explain why *B. microti* has lagged behind *B. burgdorferi* establishment on the mainland. We are now in a position to identify ecologically suitable areas for future expansion of *B. microti*. Our findings also imply that control measures such as reservoir host vaccination against *B. burgdorferi* may reduce *B. microti* transmission and therefore viability in areas that are highly endemic for *B. burgdorferi* [79]–[81]. Lastly, our integration of experimental and field data into a realistic model of *R*_0_ is a powerful approach to examine the effects of coinfection on other tick-borne pathogens as well as other pathogens transmitted by vectors other than ticks.

## Supporting Information

S1 Fig **Burden phenology of** ***Peromyscus leucopus*** **and parameters of the expected larval tick burden and expected nymphal tick burden.** The burdens represent the expected burden on a host at any time of the year starting January 1^st^. Functional forms of these representative curves are adapted from [60] and given in Equations 3 and 5.
(TIF)

S2 Fig **Phenology of the immature life states of** ***Ixodes scapularis*** **as observed in the northeastern areas of the United States.** Blue circles indicate larval and nymphal counts from field data of trapped mice for Block Island, Rhode Island and Nehantic and Pachaug State Parks, Connecticut. The radius of the circle is proportional to the number of mice with the associated burden at any given trapping session. The curves are fit using the functional forms set out in [60]. Fitted curves are shown in Fig. 3.
(TIF)

S3 Fig **Threshold curves for** ***Babesia microti*** **survival at different locations and mouse infection prevalences with** ***Borrelia burgdorferi*** **strain B348.** This figure shows differences in threshold curves, representing where *R*_0_ = 1, and associated confidence intervals that separate regions of *s_N_* and *c* where *B. microti* is expected to emerge and regions where it is expected to fade out. Threshold curves are contour curves where *R*_0_ is plotted as a function of two variables: the proportion of fed infected larvae that survive to become infectious feeding nymphs, *s_N_*, and the proportion of ticks feeding on *Peromyscus leucopus*, *c*. Plots indicate effects of location specific (Block Island and Connecticut) timing of tick activity as well as *B. burgdorferi* strain BL348 prevalence in mice (low  = 0.3 and high  = 0.8) on *R*_0_. Although the curves separate, the confidence intervals overlap, implying that coinfection with the *B. burgdorferi* strain B348 did not significantly change the expected value of *R*_0_. Differences in threshold curves for *B. burgdorferi* strain BL206 are shown in Fig. 5.
(PDF)

S1 Table ***Babesia microti*** **transmission to xenodiagnostic ticks from mice simultaneously coinfected with** ***B. microti*** **and** ***Borrelia burgdorferi*** **BL206 vs.** ***B. microti*** **alone (the reference group).** Days since infection with either or both pathogens was coded as continuous variable.
(DOCX)
