# Supplementary figures and images for: Borrelia burgdorferi Promotes the Establishment of Babesia microti in the Northeastern United States
Source: PLoS One. 2014 Dec 29;9(12):e115494. doi: 10.1371/journal.pone.0115494 (PMC4278703; doi:10.1371/journal.pone.0115494)

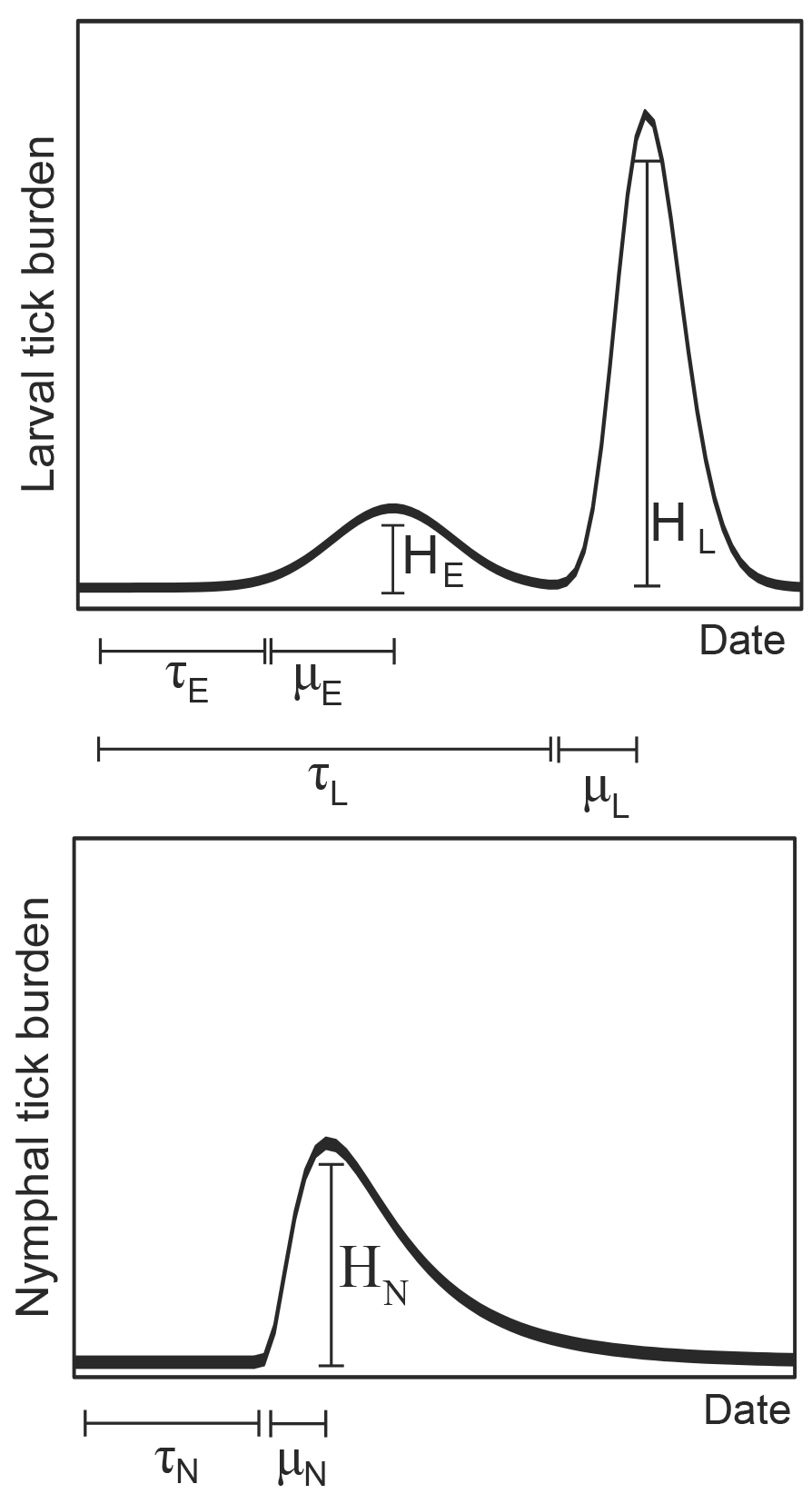

Supplement: S1 Fig — Burden phenology of Peromyscus leucopus and parameters of the expected larval tick burden and expected nymphal tick burden. The burdens represent the expected burden on a host at any time of the year starting January 1st. Functional forms of these representative curves are adapted from [60] and given in Equations 3 and 5. (TIF) [file pone.0115494.s001.tif]

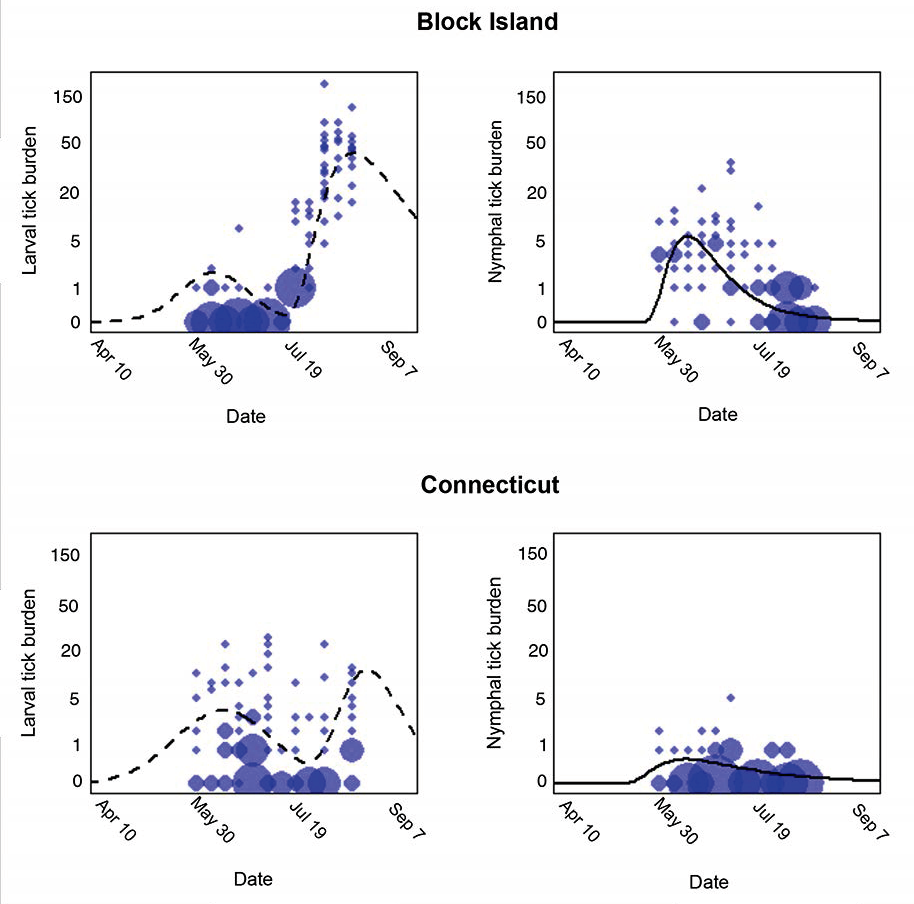

Supplement: S2 Fig — Phenology of the immature life states of Ixodes scapularis as observed in the northeastern areas of the United States. Blue circles indicate larval and nymphal counts from field data of trapped mice for Block Island, Rhode Island and Nehantic and Pachaug State Parks, Connecticut. The radius of the circle is proportional to the number of mice with the associated burden at any given trapping session. The curves are fit using the functional forms set out in [60]. Fitted curves are shown in Fig. 3. (TIF) [file pone.0115494.s002.tif]

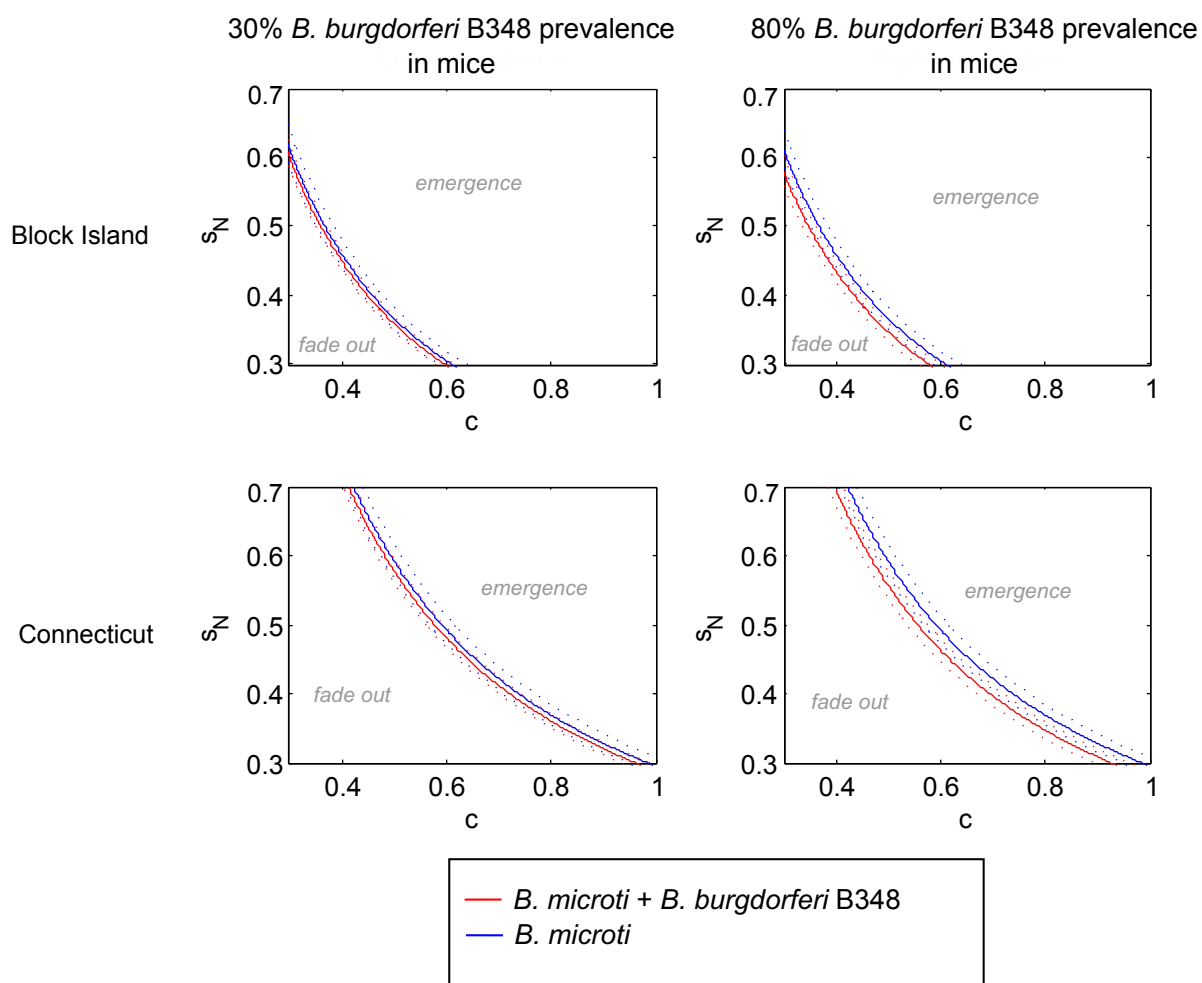

Supplement: S3 Fig — Threshold curves for Babesia microti survival at different locations and mouse infection prevalences with Borrelia burgdorferi strain B348. This figure shows differences in threshold curves, representing where R 0 = 1, and associated confidence intervals that separate regions of sN and c where B. microti is expected to emerge and regions where it is expected to fade out. Threshold curves are contour curves where R 0 is plotted as a function of two variables: the proportion of fed infected larvae that survive to become infectious feeding nymphs, sN, and the proportion of ticks feeding on Peromyscus leucopus, c. Plots indicate effects of location specific (Block Island and Connecticut) timing of tick activity as well as B. burgdorferi strain BL348 prevalence in mice (low = 0.3 and high = 0.8) on R 0. Although the curves separate, the confidence intervals overlap, implying that coinfection with the B. burgdorferi strain B348 did not significantly change the expected value of R 0. Differences in threshold curves for B. burgdorferi strain BL206 are shown in Fig. 5. (PDF) [file pone.0115494.s003.pdf]
