# Supplementary material for: Borrelia burgdorferi Promotes the Establishment of Babesia microti in the Northeastern United States
Source: PLoS One. 2014 Dec 29;9(12):e115494. doi: 10.1371/journal.pone.0115494 (PMC4278703; doi:10.1371/journal.pone.0115494)
Supplement: S1 Table — Babesia microti transmission to xenodiagnostic ticks from mice simultaneously coinfected with B. microti and Borrelia burgdorferi BL206 vs. B. microti alone (the reference group). Days since infection with either or both pathogens was coded as continuous variable. (DOCX) [file pone.0115494.s004.docx]

**Table S1.** *Babesia microti* transmission to xenodiagnostic ticks from mice simultaneously coinfected with *B. microti* and *Borrelia burgdorferi* BL206 vs. *B. microti* alone (the reference group). Days since infection with either or both pathogens was coded as continuous variable.

|  | Odds Ratio | Std. Err. | z | P>z | [95% Conf. Interval] | |
| --- | --- | --- | --- | --- | --- | --- |
| *B. microti* + *B. burgdorferi* strain BL206 | 3.73 | 2.250 | 2.18 | 0.029 | 1.14 | 12.16 |
| Days of infection | 0.95 | 0.005 | -8.03 | 0.000 | 0.940 | 0.963 |
| Constant | 1.14 | 0.328 | 0.470 | 0.641 | 0.651 | 2.007 |
